# Chronic overload of SEPT4, a parkin substrate that aggregates in Parkinson’s disease, causes behavioral alterations but not neurodegeneration in mice

**DOI:** 10.1186/1756-6606-6-35

**Published:** 2013-08-11

**Authors:** Natsumi Ageta-Ishihara, Hodaka Yamakado, Takao Morita, Satoko Hattori, Keizo Takao, Tsuyoshi Miyakawa, Ryosuke Takahashi, Makoto Kinoshita

**Affiliations:** 1 Department of Molecular Biology, Division of Biological Sciences, Nagoya University Graduate School of Science, Nagoya, Japan; 2 Department of Neurology, Kyoto University Graduate School of Medicine, Kyoto, Japan; 3 Center for Genetic Analysis of Behavior, National Institute for Physiological Sciences, Okazaki, Japan; 4 Division of Systems Medical Science, Institute for Comprehensive Medical Science, Fujita Health University, Toyoake, Japan; 5 CREST (Core Research for Evolutionary Science and Technology), JST (Japan Science and Technology Agency), Kawaguchi, Japan

**Keywords:** Parkin, Septin, Transgenic mouse, Systematic behavioral screening

## Abstract

**Background:**

In autosomal recessive early-onset Parkinsonism (PARK2), the pathogenetic process from the loss of function of a ubiquitin ligase parkin to the death of dopamine neurons remains unclear. A dominant hypothesis attributes the neurotoxicity to accumulated substrates that are exempt from parkin-mediated degradation. Parkin substrates include two septins; SEPT4/CDCrel-2 which coaggregates with α-synuclein as Lewy bodies in Parkinson’s disease, and its closest homolog SEPT5/CDCrel-1/PNUTL1 whose overload with viral vector can rapidly eliminate dopamine neurons in rats. However, chronic effects of pan-neural overload of septins have never been examined in mammals. To address this, we established a line of transgenic mice that express the largest gene product SEPT4^54kDa^ via the prion promoter in the entire brain.

**Results:**

Histological examination and biochemical quantification of SEPT4-associated proteins including α-synuclein and the dopamine transporter in the nigrostriatal dopamine neurons found no significant difference between *Sept4*^Tg/+^ and wild-type littermates. Thus, the hypothetical pathogenicity by the chronic overload of SEPT4 alone, if any, is insufficient to trigger neurodegenerative process in the mouse brain. Intriguingly, however, a systematic battery of behavioral tests revealed unexpected abnormalities in *Sept4*^Tg/+^ mice that include consistent attenuation of voluntary activities in distinct behavioral paradigms and altered social behaviors.

**Conclusions:**

Together, these data indicate that septin dysregulations commonly found in postmortem human brains with Parkinson’s disease, schizophrenia and bipolar disorders may be responsible for a subset of behavioral abnormalities in the patients.

## Background

Quantitative dysregulation of septins, a family of polymerizing scaffold proteins, is frequently associated with psychiatric disorders in humans. For instance, unbiased proteomic analyses of postmortem brains from patients with schizophrenia and bipolar disorder have uncovered excess of septin subunits including SEPT1/5/6/11 in the dorsolateral prefrontal cortices [1] and scarcity of SEPT11 in the hippocampal CA2/3 regions [2]. The neuropsychiatric significance of these phenomena is unknown.

On the other hand, occurrence of an excess of septins has been hypothesized as a critical event that accompanies a series of progressive degenerative processes in a subset of Parkinson’s disease (PD). The potential toxicity of septin overload is suggested by the following biochemical evidence and findings from animal models: Two paralogous septin subunits, SEPT5/CDCrel-1 [3] and SEPT4/CDCrel-2 (misnamed as SEPT5_v2 in [4]) are the substrates of ubiquitination by parkin, an E3 ubiquitin protein ligase whose loss of function is responsible for autosomal recessive early-onset parkinsonism (ARJP/PARK2) and a subset of sporadic PD. When the parkin function is abolished, the two septins are hypothesized to 1) accumulate with other non-septin substrates, 2) cause neuronal dysfunction, and 3) trigger neuronal death. The first step in this scenario is supported by the accumulation of SEPT5/7/8/11 in the striata of parkin-null mice [5] and of SEPT5 in dopamine neurons of transgenic mice that express a dominant negative mutant of parkin [6]. In humans, SEPT4 exceeds the normal level in the frontal cortices of postmortem brains from patients with PARK2 and in the substantia nigra from several cases of sporadic PD [4,7]. The second step is supported by the facts that SEPT5 overexpression interferes with syntaxin-mediated exocytosis from cultured cells [8,9], that parkin-null mice exhibit reduced synaptic excitability of the striatal medium spiny neurons and a mild motor coordination deficit [10], and that the dominant-negative parkin transgenic mice exhibit striatal dopamine excess and reduced voluntary movement [6]. The third step is supported by a report that dopamine neurons start to die within 15 days of the injection of SEPT5-expressing adeno-associated viral vector into rat substantia nigra [9]. Meanwhile, other reports have provided counter-evidence that dopamine neurons are morphologically intact in parkin-null mice and dominant-negative parkin transgenic mice [6,10], and that lentiviral vector-mediated expression of SEPT5 in mice (to × 1.5 of the endogenous level, co-expressed with green fluorescent protein (GFP)) did not kill neurons [11]. Thus, the presumed neurotoxicity by an excess of SEPT5 and other septins awaits further assessments with rigorous quantification of the expression level.

SEPT4, the closest paralog of SEPT5 in the mammalian genome, is unique among the septin family in several respects: First, SEPT4^54kDa^, the largest product from the mouse *Sept4* gene, contains an amino-terminal extension of ~100 amino acid residues that shares no homology with any polypeptide sequence in the database, followed by a canonical septin structure similar to SEPT5. Second, SEPT4, not SEPT5, can associate directly with α-synuclein in a certain conformation ([12,13], data not shown). Third, SEPT4, but not SEPT5, is an accessory component of α-synuclein-based aggresomes known as Lewy bodies found in PD and dementia with Lewy bodies, and as glial cytoplasmic inclusions in multiple system atrophy [14]. *Sept4*-null mice exhibit diminished dopaminergic neurotransmission due to the scarcity of a set of presynaptic proteins for dopamine turnover that include syntaxin-1, the dopamine transporter (DAT), and α-synuclein (α-Syn), all of which are co-immunoprecipitated from the striatal lysate with SEPT4 [12]. However, the effects of SEPT4 overload, either acute or chronic, have never been tested *in vivo* either with transgenic model or with viral vector-based model.

On the basis of the above background, this study critically addressed the open questions through the assessment of chronic effects of septin overload on neurodegeneration and neural dysfunctions. To this end, we have established three lines of transgenic mice that express SEPT4^54kDa^ in the brain [12], of which the one with the best breeding efficiency was used for this study (see Methods). The transgenic and nontransgenic male littermate mice were subjected to histochemical and biochemical analyses, and to an unbiased, systematic behavioral test battery as a means of sensitive functional screening.

## Results

### Generation and establishment of a transgenic mouse line that stably expresses SEPT4^54kDa^ in the brain

To assess the hypothetical harm of chronic overload of SEPT4^54kDa^ in mice, we constructed a transcription unit by inserting the coding region of the mouse *Sept4*_v1 cDNA into the mouse PrP(prion gene promoter)-polyA cassette which drives pan-neural gene expression [15]. We obtained transgenic mice by injecting the linearized transcription unit into the oocytes of C57BL/6J mice and selected founders that transmitted the transgene in Mendelian manner. After backcrossing one of the founders and the offspring with wild-type C57BL/6 mice for more than ten generations, we have established a transgenic line that consistently exhibits pan-neural expression of exogenous SEPT4^54kDa^ in addition to the endogenous *Sept4* gene products, SEPT4^54/52/48/44kDa^. In this study we analyzed male mice heterozygous for the transgene (*Sept4*^Tg/+^) with their wild-type male littermates (*Sept4*^+/+^) at the age of 5–9 months.

**Figure 1:**
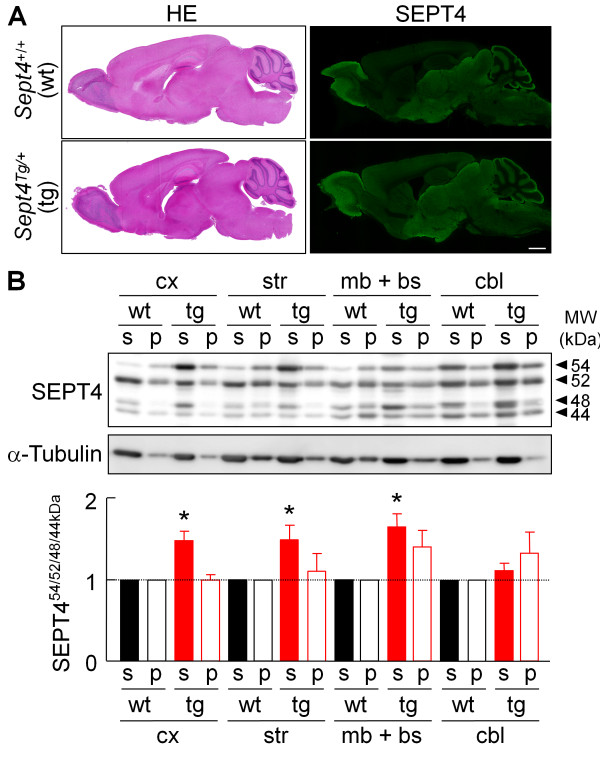
**Expression of the endogenous and transgenic SEPT4 in the mouse brain. (A)** Representative parasagittal brain sections of the wild-type (wt, *Sept4*^+/+^) and transgenic (tg, *Sept4*^Tg/+^) adult male mice stained with hematoxylin and eosin (HE, left panels), or immunostained for SEPT4 (right panels). In *Sept4*^Tg/+^ brain, the increment of SEPT4 is the most obvious where the endogenous SEPT4 expression is relatively low (*e*.*g*., the striatum, hippocampus and cerebral cortex). No recognizable anatomical and histological difference was found between *Sept4*^+/+^ and *Sept4*^Tg/+^ brains. **(B)** Quantitative immunoblot analysis of the major SEPT4 polypeptides in fractionated lysates from *Sept4*^+/+^ and *Sept4*^Tg/+^ brain regions. (Top) Representative immunoblot patterns for SEPT4 and α-tubulin (loading control) of the following brain regions; the cerebral cortex (cx), striatum (str), midbrain and brainstem (mb + bs), and cerebellum (cbl). The lysate (containing 3 μg protein) from each brain region was separated by ultracentrifugation into the supernatant/soluble (s) fraction and the pellet/insoluble (p) fraction. Immunoblot with the SEPT4 antibody (used in panel A) consistently detected four major bands of approximately 54, 52, 48 and 44 kDa, all of which were absent in *Sept4*^−/−^ mice ([12]; data not shown). The top band, containing the transgene-encoded 54 kDa isoform was consistently increased in each region of *Sept4*^Tg/+^ brain, reflecting the pan-neural expression by the prion promoter. (Bottom) Comparison of the total densitometric values of the four major SEPT4 polypeptides in the two fractions of the brain regions. The ratio of soluble SEPT4 content of *Sept4*^Tg/+^ brain over *Sept4*^+/+^ brain was roughly x 1.5, except for the cerebellum.

*Sept4*^Tg/+^ and *Sept4*^+/+^ littermates did not show recognizable differences in the whole body weight (31.0 g vs. 31.5 g; Additional file 1: Figure A1A) and in the gross and histological brain architecture (Figure 1A left, data not shown). We examined the spatial expression pattern of the transgene-derived SEPT4^54kDa^ in parasagittal and coronal brain sections by immunofluorescence with an antibody H5C2U that recognizes a carboxyl-terminal sequence shared by the major *Sept4*-derived polypeptides including SEPT4^54kDa^. The immunofluorescence signals were more intense in *Sept4*^Tg/+^ brain sections than in wild-type samples especially in the forebrain including the hippocampus and the striatum, whereas the increment was obscured by high endogenous signals in the thalamus, midbrain, and other regions (Figure 1A right).

### Quantitative expression analysis of transgenic SEPT4^54kDa^ and the endogenous SEPT4 polypeptides in brain subregions

We quantified the expression level of the endogenous and transgenic SEPT4 polypeptides in the cerebral cortex, striatum, midbrain/brainstem, and the cerebellum. Immunoblot analysis with H5C2U antibody consistently detected mainly four major bands of approximately 54, 48, 44 and 42 kDa in the soluble fractions extracted from the four brain regions (‘s’ in Figure 1B, top). Each brain region from *Sept4*^Tg/+^ mice exhibited specific abundance of SEPT4^54kDa^, corroborating the pan-neural expression of transgenic SEPT4^54kDa^ as designed. In *Sept4*^+/+^ brain, the insoluble fraction contained slightly more SEPT4^54kDa^ than the soluble fraction except for the cerebellum. By contrast, SEPT4^54kDa^ was partitioned predominantly into the soluble fraction in *Sept4*^Tg/+^ brain. Thus, we speculate that the excess SEPT4^54kDa^ is not organized into physiological higher-order heteropolymers or aggregated into inclusion bodies that cannot be extracted.

Densitometry revealed that the expression ratio of SEPT4^54kDa^ in *Sept4*^Tg/+^ brain over *Sept4*^+/+^ brain was the highest in the cerebral cortex (approximately × 8.5 in the soluble fraction), followed by the striatum (× 7), cerebellum (× 6), and midbrain and brainstem (× 5). These data appear to be compatible with the immunofluorescence results (Figure 1A right). Although the functional equivalence among the four major SEPT4 polypeptides is unknown, when their densitometric values are weighed equally, the relative SEPT4 content in *Sept4*^Tg/+^ brain was roughly × 1.5 of *Sept4*^+/+^ brain (Figure 1B, bottom). Thus, the dosage of SEPT4 in our *Sept4*^Tg/+^ mouse brain is comparable to those which would be caused by trisomies involving the *Sept4* gene locus (see Discussion).

### Chronic SEPT4^54kDa^ overload does not affect the amount and solubility of DAT, TH and α-synuclein in the striatum

**Figure 2:**
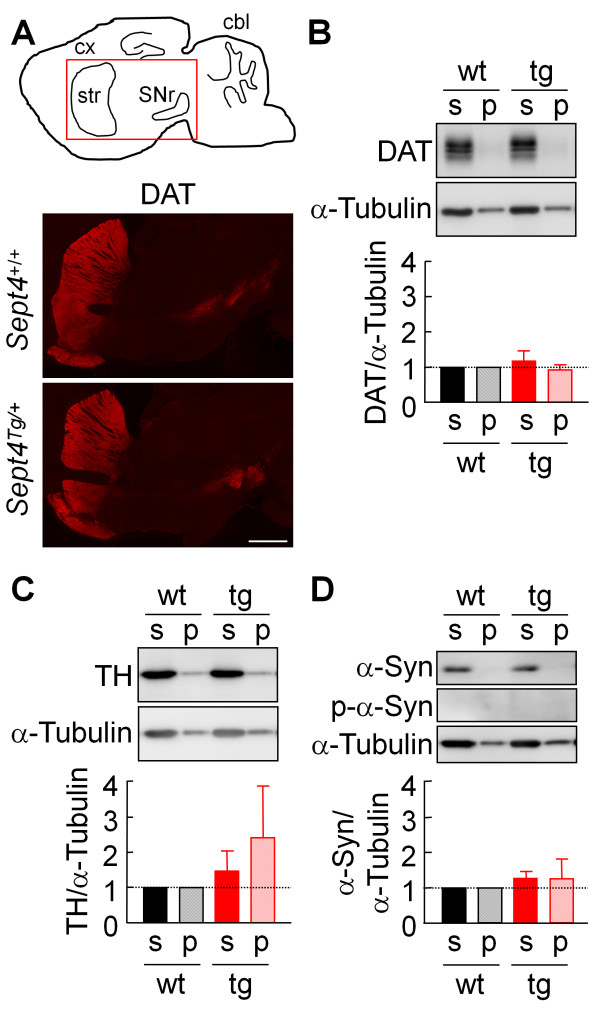
**Expression of the two major proteins for dopamine metabolism and α-synuclein in the striata of wild**-**type and *Sept4 *transgenic mice. (A)** The nigrostriatal tract (parasagittal sections) of *Sept4*^+/+^ and *Sept4*^Tg/+^ mice immunostained for the dopamine transporter (DAT). There was no recognizable morphological alteration in the nigrostriatal tract of *Sept4*^Tg/+^ mice. **(B**–**D)** Quantitative immunoblot analysis for DAT, tyrosine hydroxylase (TH), α-synuclein (α-Syn), and α-Syn phosphorylated at Ser^132^ (p-α-Syn) in the fractionated lysate of the striata of *Sept4*^+/+^ and *Sept4*^Tg/+^ mice. There were no significant quantitative changes in these proteins by the chronic overload of SEPT4.

Our previous study with *Sept4*^−/−^ mice demonstrated a significant attenuation of dopamine transmission in the striatum, partly due to the combined scarcity of DAT, α-Syn, and tyrosine hydroxylase (TH) in the axon terminals of dopamine neurons without morphological alterations [12]. *Sept4*^Tg/+^ mice did not exhibit recognizable morphological changes in the nigrostriatal tract up to light microscopic level (DAT immunofluorescence in Figure 2A, data not shown). Immunoblot analysis of the fractionated lysates of the striatum showed no significant quantitative changes in the amount and partitioning of DAT, TH, and α-Syn in *Sept4*^Tg/+^ mice (Figure 2B–D). Further, α-Syn phosphorylated at Ser^132^ (p-α-Syn), the aggregation-prone species pathognomonic for synucleinopathies [16], was not detected in *Sept4*^Tg/+^ striatum (Figure 2D). These biochemical and morphological data indicate that aberrant expression, modification or aggregation of these proteins and obvious neurodegeneration do not occur in nigrostriatal dopamine neurons in *Sept4*^Tg/+^ mice at least up to 9 months.

### *Sept4*^Tg/+^ mice exhibit attenuation of activity surges in distinct behavioral paradigms

As a means of unbiased, sensitive screening for neuropsychiatric abnormalities, *Sept4*^Tg/+^ mice were subjected to a battery of behavioral tests. Overall, male *Sept4*^Tg/+^ and *Sept4*^+/+^ littermates (n = 15 and 20) did not differ significantly in their appearance and many of the quantitative physical and behavioral indices measured (Table 1 and Additional file 1: Figure A1–A11. The raw data are accessible at the Mouse Phenotype Database, http://www.mouse-phenotype.org/). It is noteworthy that *Sept4*^Tg/+^ mice were normal in prepulse inhibition (PPI) test (Additional file 1: Figure A4) in contrast to *Sept4*^−/−^ mice which demonstrated a significant augmentation in PPI score [12].

**Table 1:** **Summary of systematic behavioral tests for *Sept4***^**Tg**/+ ^**mice in comparison with *Sept4***^−/− ^**mice**

**Tests**	**Mental/physical activities**	**Indices measured**	**Alteration from wildtype**	
		(inexhaustive)	*Sept4*^tg/+^(*tg*)	*Sept4*^−/−^(*ko*)
Table 1. Summary of systematic behavioral tests for *Sept4*^Tg/+^ (tg) mice in comparison with *Sept4*^−/−^ (ko) mice (Additional Figure A1)	General health	Body weight	→	→
		Rectal temperature	→	→
		Grip strength	→	→
		Hanging persistence	→	→
Light/dark transition test	Exploratory activity	Distance traveled in the light chamber	→	→
(Additional Figure A2)	Light avoidance	Distance traveled in the dark chamber	→	→
		Latency to the first entry to the light chamber	→	→
		Time stayed in the light chamber	→	→
		Number of transitions between chambers	→	→
Open field test	Exploratory activity	Distance traveled	→*	→
(Figure 3)	Avoidance from open space	Center time	→	→
Open field test + metamphetamine	Induced surge of exploratory activity	Distance traveled	→*	↓
(Figure 4)		Center time	→	N.D.
Elevated plus maze test	Exploratory activity	Distance traveled	→	→
(Additional Figure A3)	Height avoidance	Entries into open arms	↑	→
		Number of entries	→	→
		Time stayed on open arms	→	→
Acoustic startle response	Startle reflex to loudness	Ampliture of body motion	→	→
(Additional Figure A4A)				
Prepulse inhibition (PPI) test	Sensorimotor gating	Decrement of startle amplitude	→	↑
(Additional Figure A4B)				
Porsolt forced swim test	Despair-like behavior	Latency to immobility	→	→
(Additional Figure A5)				
Home cage monitoring	Diurnal cycle of locomotor activity	Activity level (distance traveled)	→ (basal)	→
(Figure 5)			↓(at surges)	→
Social interaction test	Social behavior, anxiety-like behavior	Distance traveled	→	→
(1 chamber, stranger pair)		Number of contacts	→	→
(Additional Figure A7)		Total duration of active contacts	→	→
		Mean contact duration	→	→
		Total duration of contacts	→	→
Social interaction test	Social behavior, anxiety-like behavior	Time spent with novel stranger	↓(Step 2)	N.D.
(3 chamber, 1–2 caged strangers)				
(Figure 6)		Distance traveled	↓(Step 2)	N.D.
Rota rod test	Motor coordination/learning	Latency to fall	→	→
(Additional Figure A6)				
Beam test	Motor coordination/learning	Moving speed	N.D.	→
Gait analysis	Mechanics of limb movement	Limb positions and timings	→ (front paw angle)	N.D.
(Additional Figure A8)			↓ (hind paw angle)	N.D.
Hot plate test	Aversive response to noxious stimulus	Latency to limb withdrawal	→	→
(Additional 1 Figure A9)				
Tail suspension test	Behavioral despair	Latency to immobility	→	→
(Additional 1 Figure A10)				
Fear conditioning test	Fear memory	Conditioning	→	→
(Additional 1 Figure A11)		Contextual testing	→	→
		Cued test with altered context	→	→

Comparative table of systematic behavioral test results with *Sept4*^Tg/+^ (tg) and wild-type littermate male mice (C57BL/6, this study) and with *Sept4*^−/−^ (ko) and wild-type littermate male mice (C57BL/6J, [12]). Symbols: →, no significant difference (p > 0.05); ↑, statistically significant increase; ↓, statistically significant decrease; in comparison with wildtype littermates. N.D., not determined.

**Figure 3:**
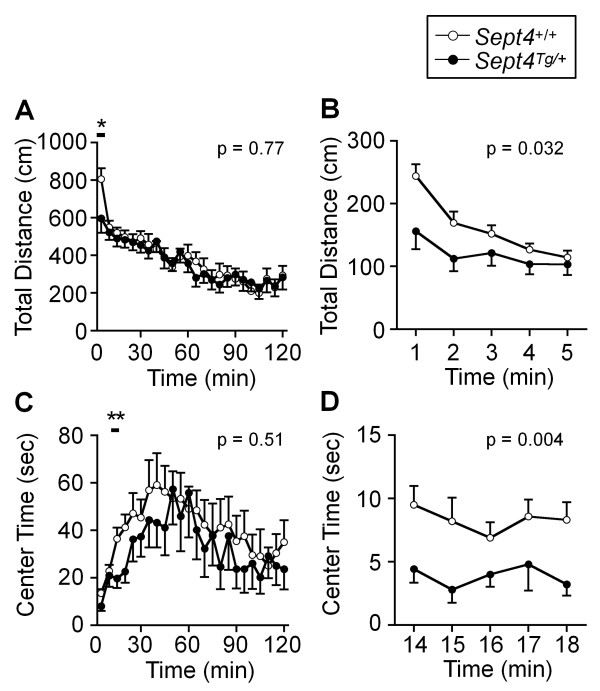
**Attenuation of the initial exploratory activity of *Sept4***^**Tg**/+ ^**mice in a novel environment. (A**, **B)** The total distance traveled during the entire session of 120 min (**A**, 5-min step) and an expansion of the initial 5 min (**B**, 1-min step) in the open field test. In the initial 5 min (bar in A), the exploratory locomotive activity of *Sept4*^Tg/+^ mice was significantly less than that of *Sept4*^+/+^ mice (*, p = 0.032, n = 20, 14). The test was conducted at the age of 6 months (cf. Figure 4). [**(A)** F_1,32_ = 0.09, p = 0.77, genotype × time interaction, F_23,736_ = 1.14, p = 0.29, **(B)** F_1,32_ = 5.04, p = 0.032, genotype × time interaction, F_4,128_ = 2.86, p = 0.026.] **(C**, **D)** The center time during the entire session (**C**, 5-min step) and an expansion of 14–18 min (**D**, 1-min step) of the open field test. The center time, another index of the exploratory activity, tended to be reduced in *Sept4*^Tg/+^ mice, which was statistically significant during an early time frame (**, p = 0.004). [**(C)** F_1,32_ = 0.45, p = 0.51, genotype × time interaction, F_23,736_ = 0.63, p = 0.91, **(D)** F_1,32_ = 9.37, p = 0.004, genotype × time interaction, F_4,128_ = 0.39, p = 0.82].

**Figure 4:**
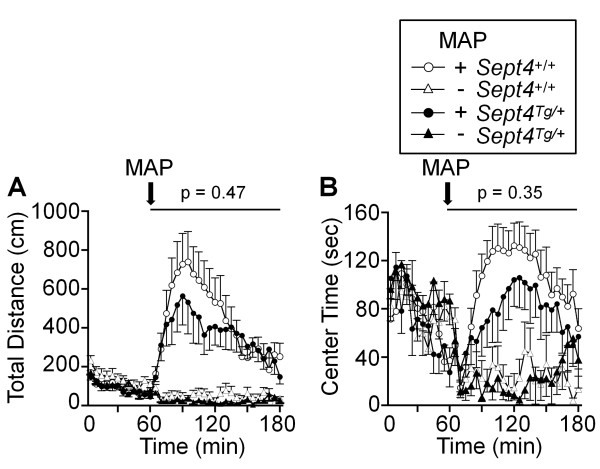
**A trend of attenuation of the metamphetamine**-**induced hyperactive responses in *Sept4***^**Tg/+ **^**mice. (A**, **B)** The responses of *Sept4*^+/+^ and *Sept4*^Tg/+^ mice to the administration of a dopaminergic psychostimulant metamphetamine (MAP) assessed in the open field test. The test was conducted with the same cohort of 9-month-old mice in the same apparatus (cf. Figure 3. Note that aging has reduced the activity level before MAP injection while extending the center time.). Each of the *Sept4*^+/+^ and *Sept4*^Tg/+^ cohorts was divided into two subgroups and intraperitoneally injected with either MAP in saline (open and closed circles, n = 10, 7) or saline (open and closed triangles, n = 9, 7) at 60 min and observed for 120 min. The metamphetamine-driven surge of the total distance traveled **(A)** and the center time **(B)** of *Sept4*^Tg/+^ mice tended to be less than those of *Sept4*^+/+^ mice. [**(A)** F_1,29_ = 0.53, p = 0.47, drug effect, F_1,29_ = 30.14, p < 0.0001, genotype × drug interaction, F_1,29_ = 0.13, p = 0.72, genotype × time interaction, F_23,667_ = 1.13, p = 0.31, drug × time interaction, F_23,667_ = 8.28, p < 0.0001, genotype × drug × time interaction, p = 0.46, **(B)** F_1,29_ = 0.92, p = 0.35, drug effect, F_1,29_ = 19.37, p = 0.0001, genotype × drug interaction, F_1,29_ = 0.62, p = 0.44, genotype × time interaction, F_23,667_ = 1.34, p = 0.13, drug × time interaction, F_23,667_ = 7.49, p < 0.0001, genotype × drug × time interaction, p = 0.21].

**Figure 5:**
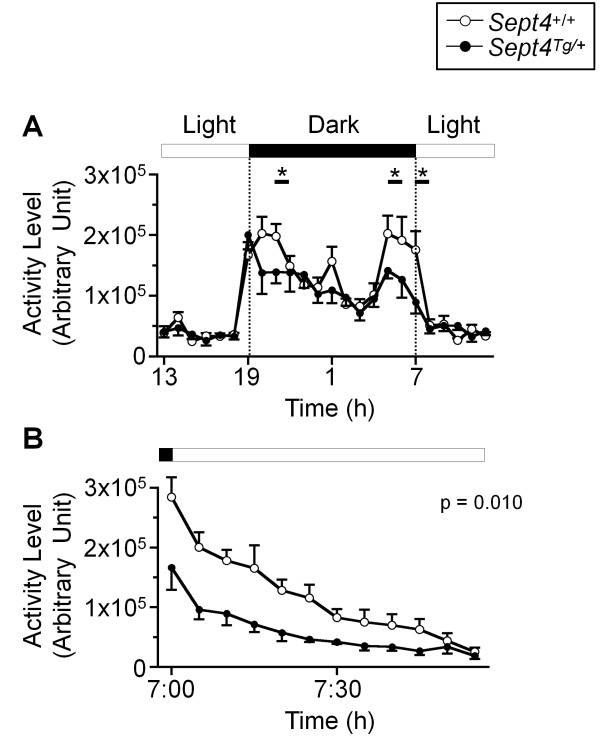
**Attenuated diurnal activity surges of *Sept4***^**Tg/+ **^**mice. (A)** Diurnal oscillation of the locomotor activity in the home cage contained three peaks in *Sept4*^+/+^ mice during the dark phase, two of which were significantly attenuated in *Sept4*^Tg/+^ mice (*, p = 0.01, n = 9, 6). **(B)** An expansion of a time block during 7:00–8:00 in 5-min step shows that the significant difference in activity lasted up to 45 min after the onset of the light phase. [F_1,14_ = 8.77, p = 0.010, genotype × time interaction, F_11,154_ = 5.26, p < 0.0001].

**Figure 6:**
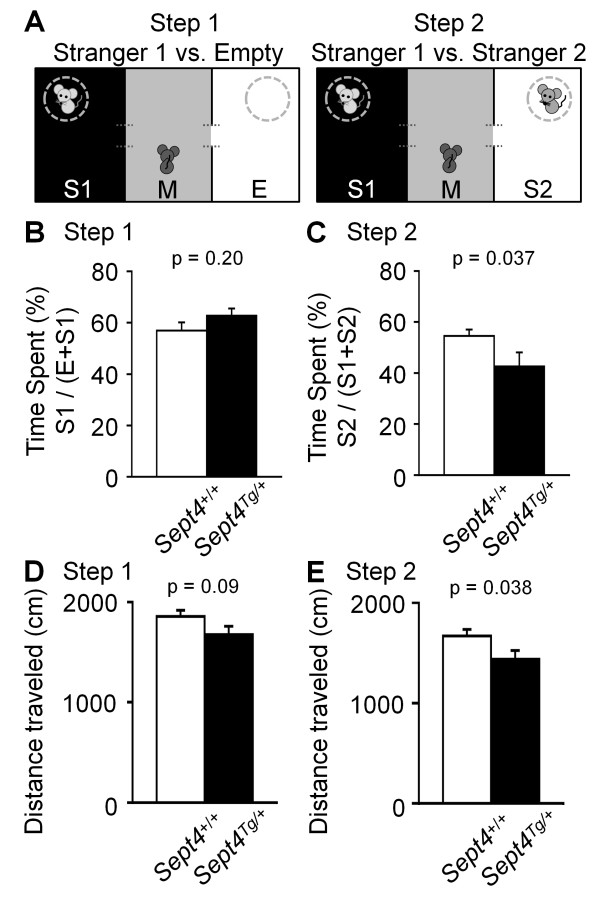
**Altered social interaction pattern of *Sept4***^**Tg/+ **^**mice. (A)** Schematic diagrams of the three-chamber sociability and social novelty preference tests [18]. (Left) The sociability test setup. Each mouse was scored for the time spent in the middle habituated chamber (M), the left chamber containing an unfamiliar C57BL/6J mouse (Stranger 1, S1) in a wire cage, or the right chamber with an empty wire cage **(E)**. (Right) The social novelty preference test following the sociability test uses the same apparatus, except for a novel unfamiliar C57BL/6J mouse (Stranger 2, S2) caged in the right chamber in addition to the now-familiar C57BL/6J mouse (Stranger 1, S1) remaining in the left cage/chamber. **(B**, **D)** There was no difference in the sociability indices (S1 over E) between the genotypes. [**(B)** F_1,32_ = 1.68, p = 0.20, **(D)** F_1,32_ = 4.75, p = 0. 037]. **(C**, **E)** In the social novelty preference test (Step2) after the sociability test (Step1), *Sept4*^Tg/+^ mice exhibited significantly less preference for S2 over S1 than *Sept4*^+/+^ mice, indicative of their reduced curiosity in the novel social stimuli and/or augmented persistence to the familiar social stimuli (n = 20, 14). [**(C)** F_1,32_ = 3.06, p = 0.09, **(E)** F_1,32_ = 4.69, p = 0. 038].

Intriguingly, however, distinct behavioral paradigms independently pointed to attenuations of voluntary locomotor activities of *Sept4*^Tg/+^ mice as follows: 1) Reduction in the distance traveled in the initial phase (p = 0.03 at 0–5 min; Figure 3A and 3B) of an open field test. 2) Reduction in the center time (another index of exploratory activity) in an early time frame of an open field test (p = 0.004 at 14–18 min; Figure 3C and D). 3) Subnormal trends in the open field test after driving the activity with methamphetamine (MAP, injected at 60 min; Figure 4A and B. Note: Reduced sample numbers by splitting groups have reduced the power of statistical analysis.) 4) Reductions in the dark phase activity surges in the home cage activity monitoring, the last of which extended to the first 45 min after the onset of the light phase (p = 0.01; Figure 5A and B). 5) Consistent reduction of locomotor activities in the social interaction tests (p = 0.038; Figure 6E; Additional file 1: Figure A7; See below). These behavioral data commonly indicate that the basal activity level of *Sept4*^Tg/+^ mice is maintained in a normal range, while their induced activity upon environmental, social, or pharmacological stimuli, or by circadian rhythm, fail to reach normal levels. It is noteworthy that the hypoactivity of *Sept4*^Tg/+^ mice is context-dependent or selective, given their normal locomotor activity in the light/dark transition and elevated plus maze tests (Additional file 1: Figure A2A and A3A).

### Unique alterations in social behaviors in *Sept4*^Tg/+^ mice

Our unbiased behavioral screening detected unexpected alterations in social activities of *Sept4*^Tg/+^ mice. In a paradigm of social interaction test with a caged stranger mouse in a three-compartment chamber [17,18], *Sept4*^Tg/+^ mice approached a caged stranger (S1) almost normally (Figure 6A, Step1; Figure 6B). However, after subsequent introduction of a second caged stranger (S2), *Sept4*^Tg/+^ mice approached S2 less frequently than their *Sept4*^+/+^ littermates did (p = 0.037; Figure 6C).

In a distinct paradigm of an open field-based social interaction test with a pair of stranger mice (Additional file 1: Figure A7A), *Sept4*^Tg/+^ mice in a chamber tended to contact less frequently than *Sept4*^+/+^ pairs did (Additional file 1: Figure A7C–E). A similar trend of reduced body contact was observed when a pair of *Sept4*^Tg/+^ mice were housed as cage-mates and monitored for a week (data not shown).

### Other behavioral alterations found in *Sept4*^Tg/+^ mice

The behavioral screening identified some other alterations in *Sept4*^Tg/+^ mice in distinct paradigms as follows: 1) In the elevated plus maze test, *Sept4*^Tg/+^ mice entered the open arms significantly more often (p = 0.02; Additional file 1: Figure A3C) and tended to stay there longer than the control mice (Additional file 1: Figure A3D). 2) In a treadmill-based, video-assisted gait pattern analysis using DigiGait [19], *Sept4*^Tg/+^ mice exhibited significantly narrower hind paw splay angle, *i*.*e*., toe-in (p = 0.001; Additional file 1: Figure A8F), without alterations in the other indices of gait mechanics.

## Discussion

### Chronic overload of SEPT4 alone is insufficient to cause neurodegeneration in mice

A widely accepted hypothesis proposed for PARK2 in humans attributes the degeneration of dopamine neurons by the loss of parkin function to the accumulation of its ubiquitination substrates that include Pael receptor/GPR37, SEPT4, SEPT5, syphilin-1, cyclin E, and aminoacyl-tRNA synthetase cofactor p38 [20]. Potential neurotoxicity of some of these substrates has been demonstrated by the viral vector-mediated rat models or genetically engineered mouse models [9,21-24]. Unlike previous studies with other substrates, this study demonstrated for the first time that mice genetically overloaded with SEPT4 do not develop obvious neurodegeneration or sensorimotor impairments at least up to 9 months of age. We regard the results as counter-evidence against potential neurotoxicity of SEPT4 overload. However, our results do not exclude the possibility that neurodegeneration triggered by other factors could be aggravated by concurrent SEPT4 overload, or that combined overload of the above substrates including SEPT4 could entail “synthetic lethality”.

### Differential suppression of dopaminergic neurotransmission and/or behavioral activity by the loss or excess of SEPT4

Our previous study demonstrated mild hypo-dopaminergic abnormalities of *Sept4*^−/−^ mice in two distinct behavioral paradigms: 1) Excess in the PPI of acoustic startle response, which was apparently normalized by apomorphine, a nonselective agonist of dopamine receptors. 2) Attenuation in the methamphetamine-driven surge of voluntary locomotion in an open field test. Consistently, SEPT4-interacting presynaptic proteins including DAT, syntaxin-1 and α-Syn were significantly reduced in nigrostriatal dopamine neurons of *Sept4*^−/−^ mice. Thus, SEPT4 is regarded as a critical subunit of septin hetero-oligomers that constitute presynaptic scaffold in dopamine neurons [12]. However, the present study has demonstrated that *Sept4*^Tg/+^ mice do not exhibit the opposite phenotype in terms of behavior (*e*.*g*., PPI and amphetamine-driven surge of locomotion) and proteome (*e*.*g*., DAT in the striatum). Thus, a chronic excess of SEPT4 alone is insufficient to trigger hyperactivity by augmenting dopaminergic neurotransmission. Unexpectedly, the SEPT4 overload entailed selective hypoactive alterations in *Sept4*^Tg/+^ mice. Although the molecular mechanism is currently unknown, we speculate that excess SEPT4 may interfere with the exocytic machinery by sequestering syntaxins as with other septin subunits [8]. This possibility is supported by the normal levels of dopamine and its major metabolites in *Sept4*^Tg/+^ striatum (data not shown).

The unexpected concordance between *Sept4*^−/−^ and *Sept4*^Tg/+^ mice in terms of voluntary activity level indicates that induced surges of dopaminergic neurotransmission require SEPT4 to be in an optimal dose range; either loss or excess could attenuate the maximal, but not the basal, voluntary activity of an animal. Similar situations may occur in human chromosome duplications involving 17q22 [25,26], in which three copies of *SEPT4* genes theoretically cause × 1.5 overload of the normal protein level. Likewise, dysregulation (mostly overload) of diverse septin subunits frequently found in the postmortem brains from patients with schizophrenia and bipolar disorder [1,2] may contribute to a subset of psychiatric symptoms.

### Could chronic overload of SEPT5 be responsible for the hyperactivity in mice?

Copy number variations involving the human chromosome locus 22q11.2 are associated with diverse developmental disorders such as DiGeorge syndrome and Emanuel syndrome, which are often accompanied by neuropsychiatric symptoms [27,28]. BAC transgenic mice that harbor a ~200 kb subregion in the locus, containing several genes including *SEPT5*, exhibited hyperactivity and other behavioral abnormalities [29,30]. On the other hand, lentiviral vector-mediated local expression of SEPT5 in the dorsal hippocampi or basolateral amygdalae did not alter the voluntary locomotor activity of mice in an open field test [11]. Given the structural similarity between SEPT5 and SEPT4, we speculate that chronic pan-neural expression of SEPT5 would entail a phenocopy of *Sept4*^Tg/+^ mice, with an attenuated voluntary locomotor activity via the aforementioned interference on the exocytic machinery [31]. If so, the hyperactivity of the BAC transgenic mice should be ascribed to a gene(s) other than *SEPT5*. This hypothesis is to be critically tested either by chronic overload of SEPT5 alone in *Sept5* transgenic mice, or by reducing the dosage of SEPT5 from the BAC transgenic mice by intercrossing them with *Sept5*-null mice [32,33].

### Involvement of septins in social behaviors

Positive correlation between the levels of septin expression and social interaction has been suggested by the following pieces of evidence: 1) Scarcity of SEPT5 in the presynaptic varicosities in the striata of rats that had been reared in social isolation [34]. 2) Reduced social interaction in *Sept5*-null mice (Note: The phenotype is influenced by genetic background) [35]. 3) Augmented social interaction after the local expression of SEPT5 in the basolateral amygdalae [11]. In contrast, stranger pairs of *Sept4*^Tg/+^ mice tended to contact less frequently than those of *Sept4*^+/+^ mice did (Additional file 1: Figure A7). Further, *Sept4*^Tg/+^ mice approached novel stranger (S2) less frequently than *Sept4*^+/+^ mice (Figure 6). Although the underlying mechanisms are currently unknown, the unique phenotype of *Sept4*^Tg/+^ mice may indicate their attenuated novelty-seeking behaviors, as with the aforementioned reduction of exploratory activities, which commonly depend on dopaminergic neurotransmission.

## Conclusions

As opposed to the prevalent but unproven notion that accumulation of parkin substrates could cause neurodegeneration, our results would make a case against a hypothesis that chronic overload of SEPT4 as a major neurotoxic factor in Parkinson’s disease. However, as the first genetic model of pure septin overload, *Sept4*^Tg/+^ mice with the intriguing behavioral phenotype should provide a partial model for human chromosome duplications involving 17q22, and for psychiatric symptoms in patients with septin dysregulations that frequently accompany schizophrenia and bipolar disorder.

## Materials and methods

### Generation and establishment of a line of transgenic mice that overexpress SEPT4^54kDa^

To generate transgenic mice that overexpress mouse SEPT4^54kDa^ in the brain, mouse *Sept4*_v1 cDNA was inserted between 5’- and 3’-untranslated regions of the mouse prion protein transcription unit (MoPrP) containing the brain-specific promoter [15]. The linearized MoPrP-*Sept4* transcription unit was injected into C57BL/6J mouse oocytes, with or without co-injection of a linearized CMV-driven expression plasmid for GFP as a transgenic marker. Genotyping was determined by polymerase chain reaction for an artificial sequence near the MoPrP-*Sept4* junction. Three transgenic lines that expressed comparable levels of exogenous SEPT4^54kDa^ in the brain, with or without systemic GFP expression, had been established. The two lines of MoPrP-*Sept4*; CMV-*GFP* were previously used for transgenic rescue experiments of *Sept4*^−/−^ mice [12]. In the present study, the third line without GFP had been selected for robust fertility, and backcrossed with C57BL/6 mice for more than 10 generations, when consistent Mendelian transmission of the transgene was observed. *Sept4*^Tg/+^ and control wild-type (*Sept4*^+/+^) mice for behavioral analyses were bred as littermates from *Sept4*^Tg/+^ and wild-type pairs. The third transgenic line was deposited to RIKEN Bioresource Center (RBRC03800).

### Animals and experimental design

All animal procedures for breeding, behavioral tests, and tissue sampling were conducted in accordance with the guidelines of the Animal Use and Care Committees of Kyoto University, Nagoya University, and the National Institute for Physiological Sciences. All comparisons were made between male littermates to minimize confounding effects of different genetic background and environment. All behavioral tests were conducted when the mice were 5–9 months old as described previously [36,37]. Mice were group housed (4 mice per cage) in a specific pathogen-free room kept at 23 ± 2°C with a 12-hr light/dark cycle (lights on at 7 a.m.) with access to food and water. Behavioral testing was conducted between 9 a.m. and 6 p.m. except for the home cage social interaction monitoring. After each test, all apparatuses were cleaned with sodium hypochlorite solution to minimize odor.

The behavioral tests were conducted in the following order: general health and neurological screening (including body weight and temperature measurements, grip strength test, and righting, whisker touch, and ear twitch reflexes), wire hang test, light/dark transition test, open field test, elevated plus maze test, rotarod test, hot plate test, one-chamber social interaction test, Crawley's sociability and preference for social novelty test, acoustic startle response/PPI test, Porsolt forced swim test, gait analysis, fear conditioning test, tail suspension test, long-term monitoring of locomotion and social interaction in home cage, and open field test with methamphetamine. Intervals between tests were >24 h.

### Neuromuscular strength tests

Neuromuscular strength was assessed with the forelimb grip strength test and wire hang test. Forelimb grip strength was measured by pulling a mouse in the tail while its forepaws hung on to a wire grid attached to a spring balance (O’Hara & Co.). The peak force (N) until each mouse released the grid was measured three times, and the greatest value was statistically analyzed. In the wire hang test, a wire mesh with a mouse on top was slowly inverted and the latency to fall was measured with a cut-off time of 60 s.

### Light/dark transition test

The apparatus had a pair of differentially illuminated (390 lux vs. 2 lux) chambers (21 × 41 × 25 cm) connected with a door in the middle. Each mouse was released in the dark chamber, and image data were acquired from the top with a CCD camera for 10 min. The latency until the first entry into the light chamber, the time spent in each chamber, the number of transitions, and the total distance traveled were automatically measured using ImageLD software (see Image analysis) [36].

### Open field test

Voluntary locomotor activity was measured in an open field test. Each mouse was placed in the center of the open field apparatus (40 × 40 × 30 cm; Accuscan Instruments) illuminated at 100 lux. The following indices were monitored for 120 min; total distance traveled, rearing (labeled as vertical activity) measured by counting the number of photobeam interruptions, time spent in the center area of 20 × 20 cm, and the beam-break counts (labeled as stereotyped behaviors). After the completion of the battery of behavioral tests, the mice of each genotype randomly split into two groups were assessed likewise from 1 h before through to 2 h after the intraperitoneal injection of saline with or without methamphetamine (MAP, 3 μg/g body weight).

### Elevated plus maze test

The apparatus had two open arms (25 × 5 cm, with 3-mm-high plastic ledges) and two closed arms (25 × 5 cm, with 15-cm-high transparent walls) interconnected via a central crossing (5 × 5 cm), which was set at 55 cm-height and illuminated at 100 lux. The numbers of entries into, and the time spent in the open and enclosed arms, were recorded for 10 min. Image data were acquired from the top with a CCD camera, and the number of entries into and the time spent in the open/closed arms, and total distance traveled (cm) were measured automatically using ImageEP software (see Image analysis) [38].

### Rota-rod test

Motor coordination and motor leaning were tested by measuring the survival duration of keeping pace with a 3-cm-thick rotating rod which was accelerated from 4 to 40 rpm over 5 min (Rota-rod, UGO Basile). Each mouse was subjected to 6 trials over 2 days.

### Hot plate test

Sensitivity and responsiveness to a noxious stimulus was represented by the latency to the first response after placing mice on 55°C-plate (Columbus Instruments).

### One-chamber social interaction test

The positions of two mice placed in a novel chamber (40 × 40 × 30 cm) were monitored from the top at 1 frame/sec. Their horizontal distance traveled and the number of contacts were measured automatically using ImageSI software (see Image analysis).

### Crawley's test for sociability and preference for social novelty

The test was conducted as described [17,18]. The apparatus had three chambers (20 × 40 × 22 cm) separated by two transparent partitions each with an opening (5 × 3 cm), and a lid with an infrared CCD camera. A male mouse (8–12 weeks old C57BL/6J, termed Stranger 1) that had no prior contact with the subject mice was enclosed in a cylinder cage (9 cm ϕ, set in the left chamber) that allowed nose contacts. The subject mouse was released in the middle chamber and allowed to explore for 10 min, while the time spent in each chamber and within 5 cm from each cage was measured automatically using ImageCSI software (see Image analysis). Subsequently, another unfamiliar mouse (Stranger 2) was placed in another cylinder cage (in the right chamber) and monitored likewise for another 10 min. One-way ANOVA was applied for the statistical analysis for the comparison within each genotype (*e*.*g*., Empty vs. Stranger 1, and Stranger 1 vs. Stranger 2).

### Acoustic startle response and PPI test

A mouse restrained in a cylinder was placed in the chamber of a startle reflex measurement system (O'Hara & Co.) with 70 dB background white noise. After 10 min, the mouse’s startle response to a startle stimulus (40 ms of 110 or 120 dB white noise) was measured by a motion sensor for 140 ms (temporal resolution, 1 ms). A test session was a random sequence of four trials with a prepulse stimulus (40 ms of 74 or 78 dB white noise 100 ms prior to a startle stimulus) and two without. Six blocks of 6 trial types were presented in pseudorandom order with the average inter-trial interval of 15 s.

### Porsolt forced swim test

Each mouse was placed in 7.5-cm-deep water at 23°C in an acrylic cylinder (10 cm ϕ), and the duration of the struggle for evacuation was measured up to 10 min automatically using ImagePS software (see Image analysis).

### Gait analysis

Automated gait analysis on a treadmill was conducted with DigiGait (Mouse Specifics Inc.). Each mouse was forced to walk on a treadmill moving at 24 cm/sec, when the mouse body movement and paw footprints were recorded at 150 frames/sec with a CCD camera underneath the treadmill. Multiple quantitative parameters (length, width and timing of the strides, paw angle etc.) were extracted from the time-lapse images with bundled software.

### Contextual and cued fear conditioning test

Each mouse was exposed to a test chamber (26 × 34 × 33 cm) for 2 min, then to three pairs of a cue (55 dB white noise for 30 sec) each followed by a mild footshock (0.3 mA for 2 sec), repeated at 2-min intervals. For the context testing after 1 or 8 days, freezing was measured in the same chamber. For the cued testing in a distinct spatial context after 1 or 8 days, freezing after the noise was measured in a triangular chamber (35 × 35 × 41 cm) in a different room. The control of the stimuli, image acquisition at 1 frame/sec from the top, and image analysis were done automatically with ImageFZ software (see Image analysis). The criterion of freezing was defined when the difference of binarized mouse areas from two consecutive frames was below 10 pixels and lasted for 2 sec or longer.

### Tail suspension test

The behavior of each mouse suspended by the tail at a height of 30 cm was recorded for 10 min and analyzed using ImageTS software (see Image analysis).

### Monitoring voluntary activity in the home cage

The position of each mouse housed alone in a cage was monitored from the top continuously for a week. The total distance traveled was measured automatically using ImageHA software (see Image analysis).

### Image analysis

The application programs for behavioral data acquisition and analysis (ImageLD, EP, CSI, PS, FZ, TS, HA) were created on the platform of NIH Image (http://rsb.info.nih.gov/nih-image/) and ImageJ (http://rsb.info.nih.gov/ij/) by TM.

### Antibodies

SEPT4 antibody (H5C2) had been raised against the carboxyl terminus of mouse SEPT4^54kDa^/SEPT4_v1 [14], and the specificity had been confirmed by the absence of the major signals in *Sept4*^−/−^ mouse brain sections [12]. Commercial antibodies were used for the following proteins: TH and DAT (AB1542 and MAB369, Chemicon/Millipore), α-synuclein (Clone 42, BD), pSer^129^α-synuclein (Wako), and α-tubulin (Sigma). For immunofluorescence, Alexa 488- or 594-conjugated secondary antibodies against rabbit or mouse IgGs (Molecular Probes/Invitrogen) were used. The band patterns in immunoblot and the staining pattern in immunofluorescence were consistent with previous independent studies with these antibodies [39].

### Histochemistry, immunofluorescence and microscopy

Mouse brains were dissected after deep anesthesia with sodium pentobarbital (50 μg/g, i.p.) with transcardial perfusion with 0.01 M phosphate-buffered saline (PBS). For histological analysis, mice were further perfused with 4% paraformaldehyde and 0.2% picric acid in 0.1 M PBS. Hematoxylin/eosin stain and immunofluorescence was performed on formaldehyde-fixed 30 μm-thick floating brain sections, as previously described [40]. Specimens were observed with a 20× objective lens (UPlanApo, NA 0.7, Olympus) and a CCD camera (Retiga EXi, Q Imaging) attached to an upright microscope (BX-60, Olympus). A CCD microscope (BZ-9000, Keyence) with a 40× objective lens (Plan Apo, NA 1.3, Nikon) was used for semi-automatic scanning and tiling of bright-field and fluorescence images.

### Biochemical fractionation of brain subregions by graded extraction

The cerebral cortex, striatum, midbrain and brainstem, and cerebellum from *Sept4*^Tg/+^ and wild-type littermate mice (3–7 m.o.) were dissected, weighed, homogenized by sonication in 3 ml/g of buffer A (10 mM Tris–HCl at pH 7.5, 0.15 M NaCl, 1% Triton X-100, and protease inhibitors). The supernatant after centrifugation at 20,400 × g at 4°C for 0.5 h was labeled as soluble fraction. The washed pellet was dissolved with sonication in buffer B (50 mM Tris–HCl pH 6.8, 2% SDS, 10% glycerol), whose final volume was adjusted to that of the cognate soluble fraction, and was labeled as pellet/insoluble fraction.

### Immunoblotting, signal detection, densitometry

The protein content in each fraction was measured and incubated with Laemmli buffer. Each soluble fraction was loaded with the equivalent amount of the cognate pellet/insoluble fraction in an adjacent lane of 8% or 10% polyacrylamide gel. Polypeptides resolved by electrophoresis were transferred onto reinforced nitrocellulose membranes (Whatman). The membranes were blocked with 5% skim milk in Tris-buffered saline (TBS; 100 mM Tris–HCl at pH 7.4, 150 mM NaCl) containing 0.05% Tween-20 and incubated serially with the primary antibodies and anti-rabbit or -mouse IgG conjugated with horseradish peroxidase (Jackson ImmunoResearch). After extensive wash with TBS plus 0.05% Tween-20, chemiluminescence detection and densitometry were conducted with ECL-Plus reagent (PerkinElmer) and an image analyzer LAS 4000 mini with MultiGauge software (GE).

### Statistical analysis

Quantitative data were expressed as mean ± SEM. For statistical analyses, either two tailed t-test or ANOVA (one-way, two-way repeated measures, and repeated measures with two factors) were applied using StatView (SAS institute). F and p values represent the effects of genotype unless otherwise noted (Figures 3–6).

## Abbreviations

α-Syn: α-synuclein; bs: Brainstem; cbl: Cerebellum; cx: Cerebral cortex; DAT: Dopamine transporter; HE: Hematoxylin and Eosin; MAP: Methamphetamine; Mb: Midbrain; Str: Striatum; Tg: Transgenic; TH: Tyrosine hydroxylase; Wt: Wild-type.

## Competing interests

The authors declare no competing financial interests.

## Authors’ contributions

TMO mainly conducted the behavioral tests under the supervision of SH, KT, and TM. NA-I and HY conducted the biochemical, histological and statistical analyses under the supervision of RT and MK. MK designed the study and wrote the manuscript. All authors read and approved the manuscript.

## Supplementary Material

Additional file 1 **Overall, quantitative data were expressed as mean ± SEM, and either one-way or two-way repeated measures ANOVA was applied for statistical analyses.** F and p values represent the effects of genotype unless otherwise noted. **Figure A1.** Normal body weight, rectal temperature, and muscle strength of *Sept4*^Tg/+^ mice. **Figure A2.** Normal locomotor activity of *Sept4*^Tg/+^ mice in the light/dark transition test. **Figure A3.** Reduced anxiety-like behavior of *Sept4*^Tg/+^ mice in the elevated plus maze test. **Figure A4.** Normal acoustic startle response and normal sensorimotor gating of *Sept4*^Tg/+^ mice in the prepulse inhibition (PPI) test. **Figure A5.** Normal depression-like behavior of *Sept4*^Tg/+^ mice in Porsolt forced swim test. **Figure A6.** Normal motor coordination and motor learning of *Sept4*^Tg/+^ mice in the rotating rod test. **Figure A7.** Reduced physical contact between pairs of *Sept4*^Tg/+^ mice in an open field. **Figure A8.** Reduced hind paw splay angle of *Sept4*^Tg/+^ mice in the gait analysis. **Figure A9.** Normal responsiveness of *Sept4*^Tg/+^ mice toward noxious stimuli in the hot plate test. **Figure A10.** Normal depression-like behavior of *Sept4*^Tg/+^ mice in the tail suspension test. **Figure A11.** Normal contextual and cued fear conditioning of *Sept4*^Tg/+^ mice. Click here for file
