# Supplementary material for: Chronic overload of SEPT4, a parkin substrate that aggregates in Parkinson’s disease, causes behavioral alterations but not neurodegeneration in mice
Source: Mol Brain. 2013 Aug 11;6:35. doi: 10.1186/1756-6606-6-35 (PMC3751304; doi:10.1186/1756-6606-6-35)
Supplement: Additional file 1 — Overall, quantitative data were expressed as mean ± SEM, and either one-way or two-way repeated measures ANOVA was applied for statistical analyses. F and p values represent the effects of genotype unless otherwise noted. Figure A1. Normal body weight, rectal temperature, and muscle strength of Sept4Tg/+ mice. Figure A2. Normal locomotor activity of Sept4Tg/+ mice in the light/dark transition test. Figure A3. Reduced anxiety-like behavior of Sept4Tg/+ mice in the elevated plus maze test. Figure A4. Normal acoustic startle response and normal sensorimotor gating of Sept4Tg/+ mice in the prepulse inhibition (PPI) test. Figure A5. Normal depression-like behavior of Sept4Tg/+ mice in Porsolt forced swim test. Figure A6. Normal motor coordination and motor learning of Sept4Tg/+ mice in the rotating rod test. Figure A7. Reduced physical contact between pairs of Sept4Tg/+ mice in an open field. Figure A8. Reduced hind paw splay angle of Sept4Tg/+ mice in the gait analysis. Figure A9. Normal responsiveness of Sept4Tg/+ mice toward noxious stimuli in the hot plate test. Figure A10. Normal depression-like behavior of Sept4Tg/+ mice in the tail suspension test. Figure A11. Normal contextual and cued fear conditioning of Sept4Tg/+ mice. [file 1756-6606-6-35-S1.pdf]

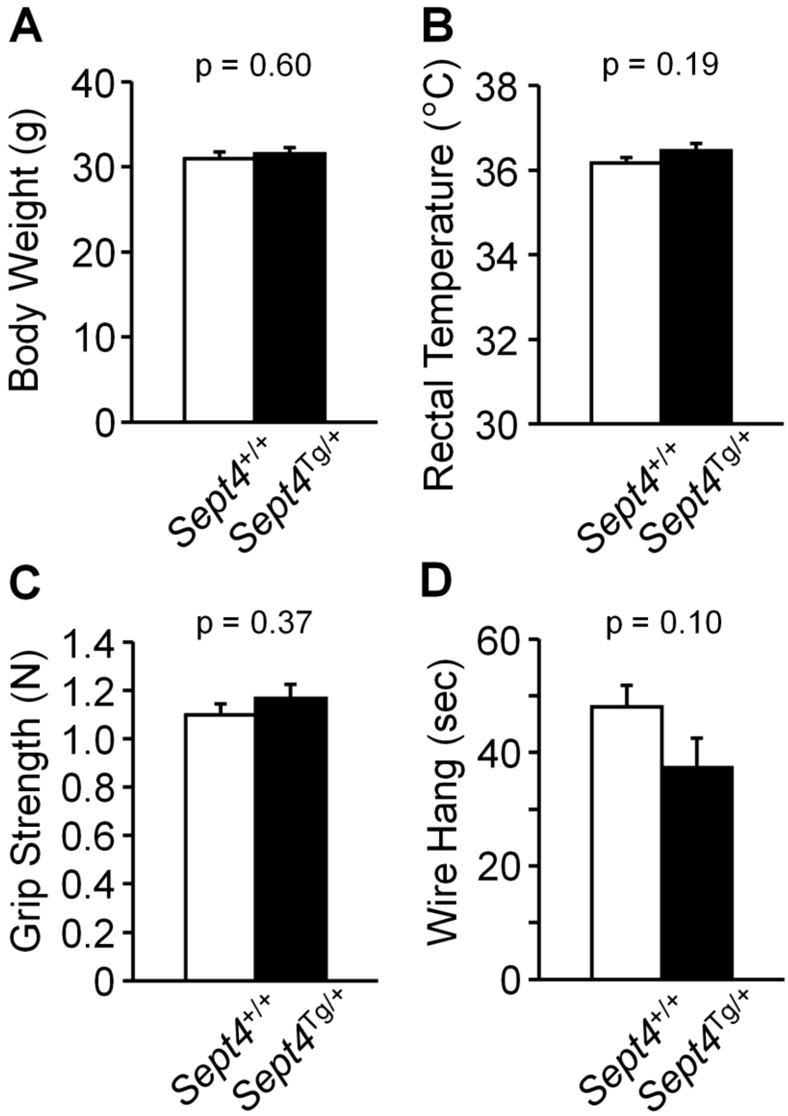

Additional Figure A1. Normal body weight, rectal temperature, and muscle strength of *Sept4*$^{Tg/+}$ mice

(A) Body weight [$F_{1,33}$=0.28, p=0.60], (B) rectal temperature [$F_{1,33}$=0.19, p=1.79], (C) grip strength [$F_{1,33}$=0.84, p=0.37], and (D) wire hang latency [$F_{1,33}$=2.88, p=0.10] of *Sept4*$^{+/+}$ and *Sept4*$^{Tg/+}$ mice (n=20, 15).

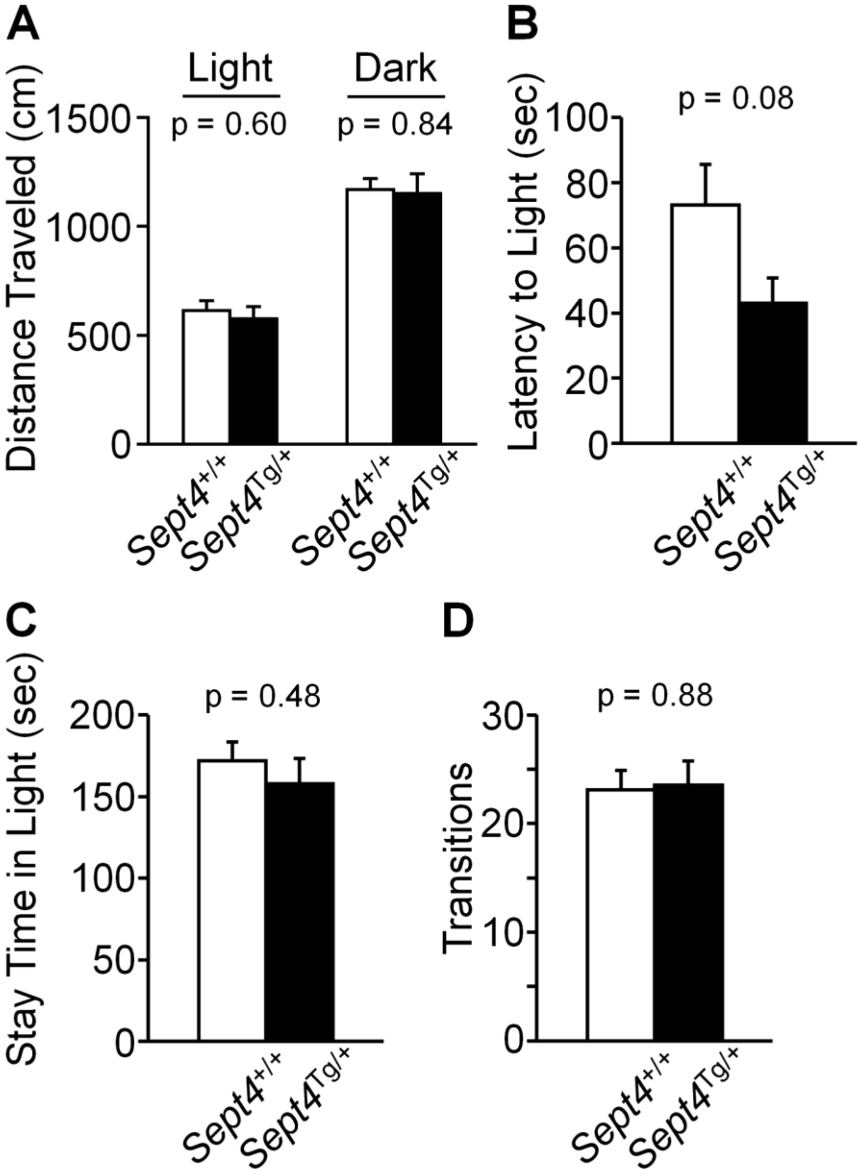

Additional Figure A2. Normal locomotor activity of *Sept4*$^{Tg/+}$ mice in the light/dark transition test

(A) Distance traveled in the light [$F_{1,31}$=0.28, p=0.60] and dark chambers [$F_{1,31}$=0.04, p=0.84], (B) latency until the first entry into the light chamber [$F_{1,31}$=3.32, p=0.08], (C) time spent in the light chamber [$F_{1,31}$=0.52, p=0.48], and (D) number of transitions across the light/dark border [$F_{1,31}$=0.02, p=0.88] of *Sept4*$^{+/+}$ and *Sept4*$^{Tg/+}$ mice (n=20, 13).

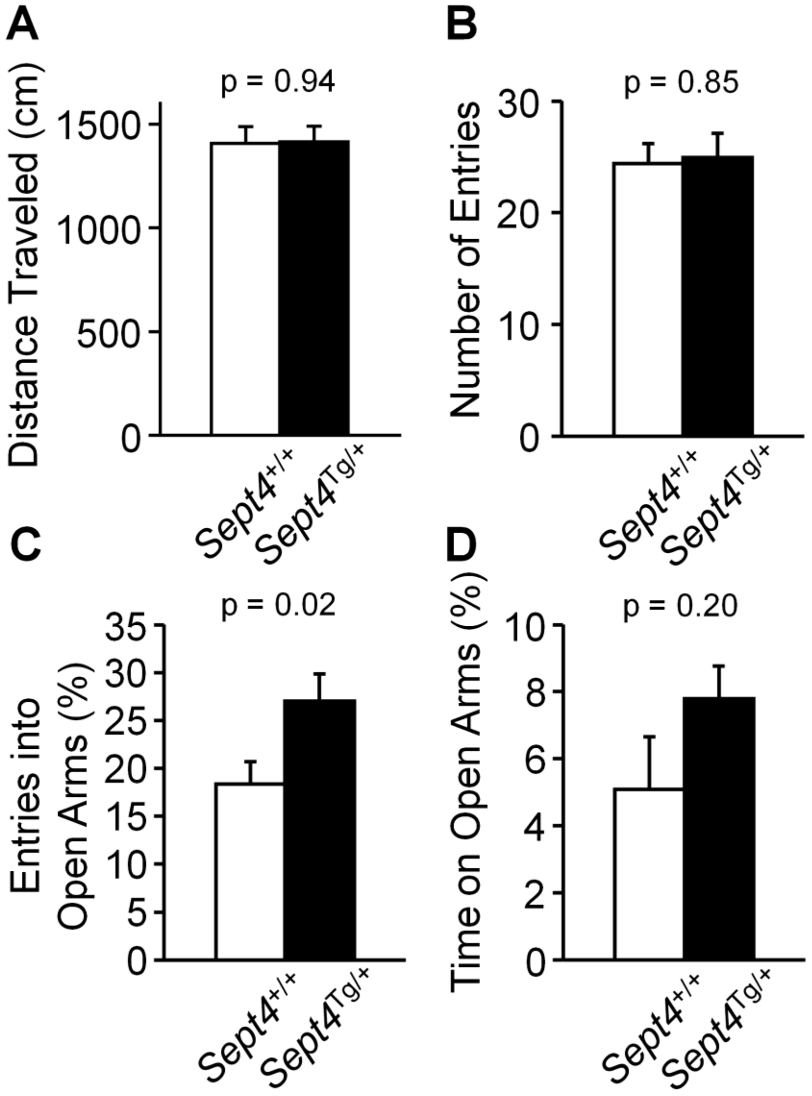

Additional Figure A3. Reduced anxiety-like behavior of *Sept4*$^{Tg/+}$ mice in the elevated plus maze test

(A) Distance traveled [$F_{1,32}$=0.01, p=0.94], (B) total number of entries into open and closed arms [$F_{1,32}$=0.04, p=0.85], (C) percentage of entries into open arms [$F_{1,32}$=5.60, p=0.02], and (D) percentage of stay time on open arms [$F_{1,32}$=1.74, p=0.20] of *Sept4*$^{+/+}$ and *Sept4*$^{Tg/+}$ mice (n=20, 15).

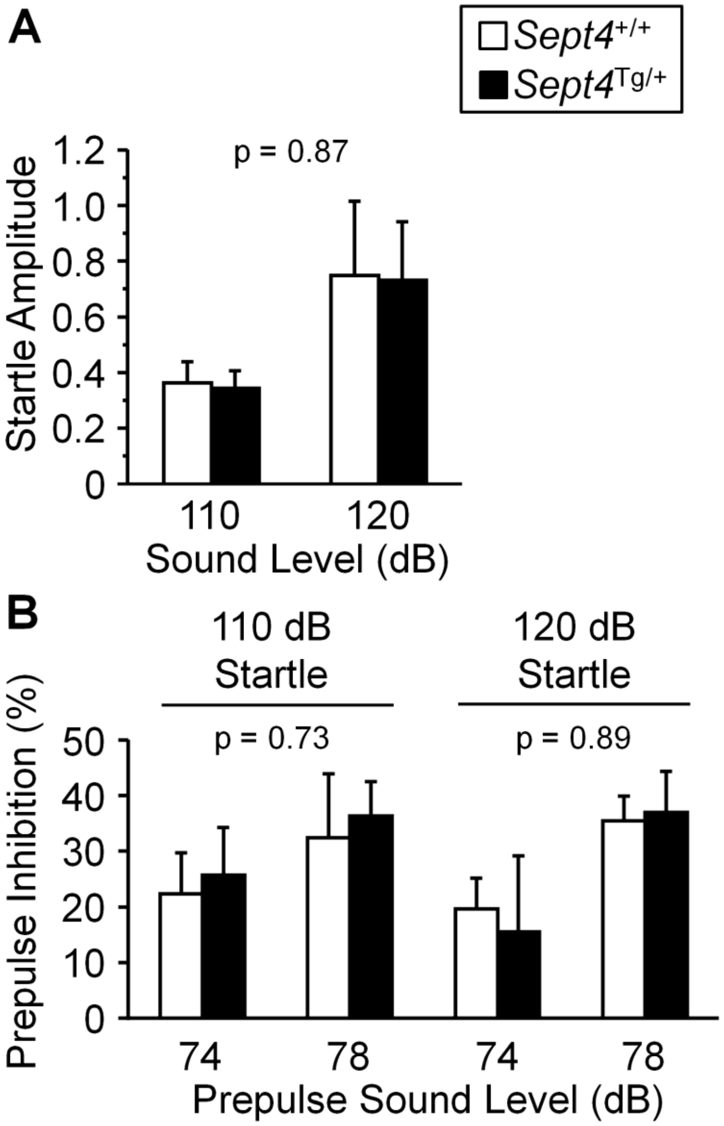

Additional Figure A4. Normal acoustic startle response and normal sensorimotor gating of *Sept4*$^{Tg/+}$ mice in the prepulse inhibition (PPI) test

(A) Startle amplitude (arbitrary unit) against acoustic stimuli of two distinct loudness [$F_{1,32}$=0.03, p=0.87, genotype x sound interaction, $F_{1,32}$=0.001, p=0.97], and (B) percent reduction of startle amplitude in the presence of a preceding acoustic stimulus (prepulse) [110 dB; $F_{1,32}$=0.12, p=0.73, genotype x sound interaction, $F_{1,32}$=0.001, p=0.98. 120 dB; $F_{1,32}$=0.02, p=0.89, genotype x sound interaction, $F_{1,32}$=0.26, p=0.61], of *Sept4*$^{+/+}$ and *Sept4*$^{Tg/+}$ mice (n=20, 14).

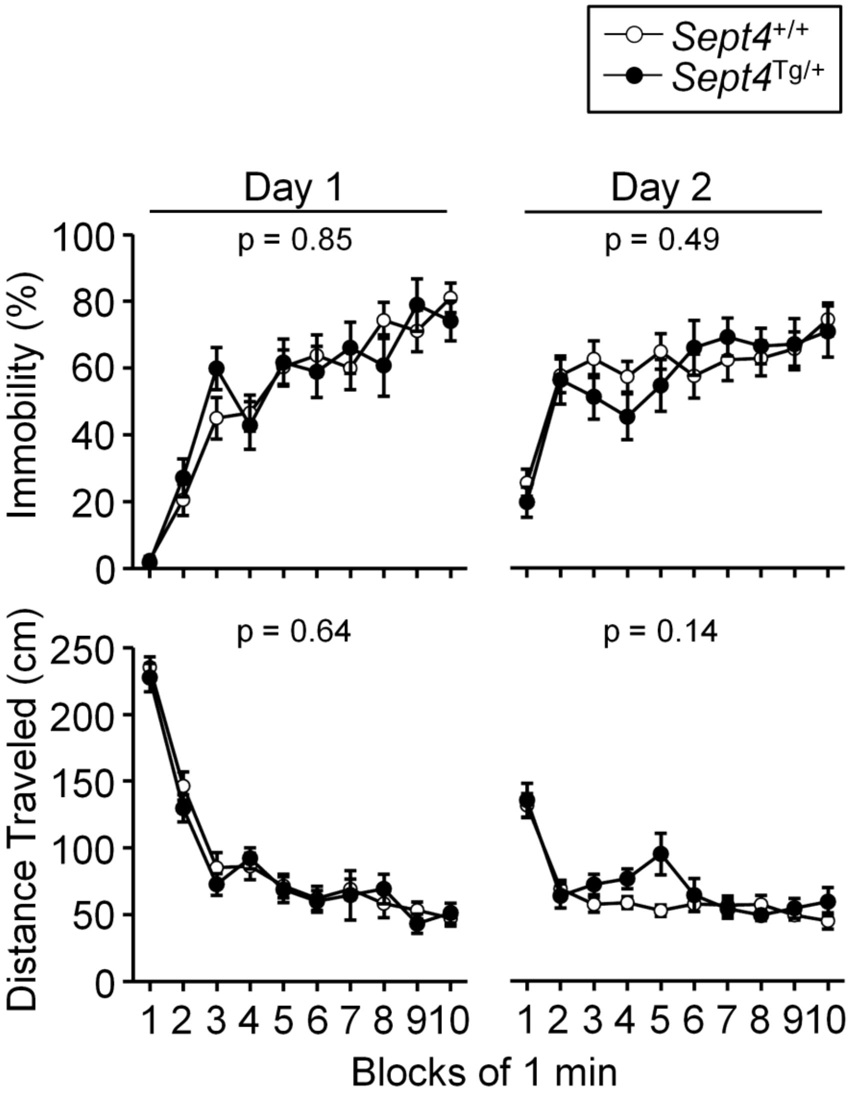

Additional Figure A5. Normal depression-like behavior of *Sept4*$^{Tg/+}$ mice in Porsolt forced swim test

(Top) Percent immobility [Day 1; $F_{1,31}$=0.04, p=0.85, genotype x block interaction, $F_{9,279}$=1.04, p=0.41, Day 2; $F_{1,31}$=0.48, p=0.49, genotype x block interaction, $F_{9,279}$=0.83, p=0.59], and (bottom) distance traveled [Day 1; $F_{1,31}$=0.22, p=0.64, genotype x block interaction, $F_{9,279}$=0.52, p=0.86, Day 2; $F_{1,31}$=2.33, p=0.14, genotype x block interaction, $F_{9,279}$=2.18, p=0.02], of *Sept4*$^{+/+}$ and *Sept4*$^{Tg/+}$ mice floating in water (n=19, 14).

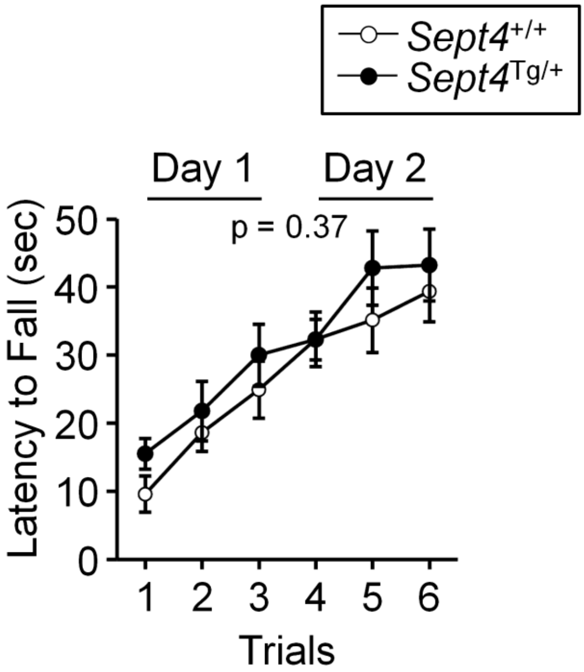

Additional Figure A6. Normal motor coordination and motor learning of *Sept4*$^{Tg/+}$ mice in the rotating rod test

The durations in which *Sept4*$^{+/+}$ and *Sept4*$^{Tg/+}$ mice (n=20, 14) kept pace with a rotating rod with a constant acceleration comparably increased during 6 trials in two days [$F_{1,32}$=0.84, p=0.37, genotype x trial interaction, $F_{5,160}$=0.46, p=0.81]. *Sept4*$^{Tg/+}$ mice never fell short of *Sept4*$^{+/+}$ mice at any trial.

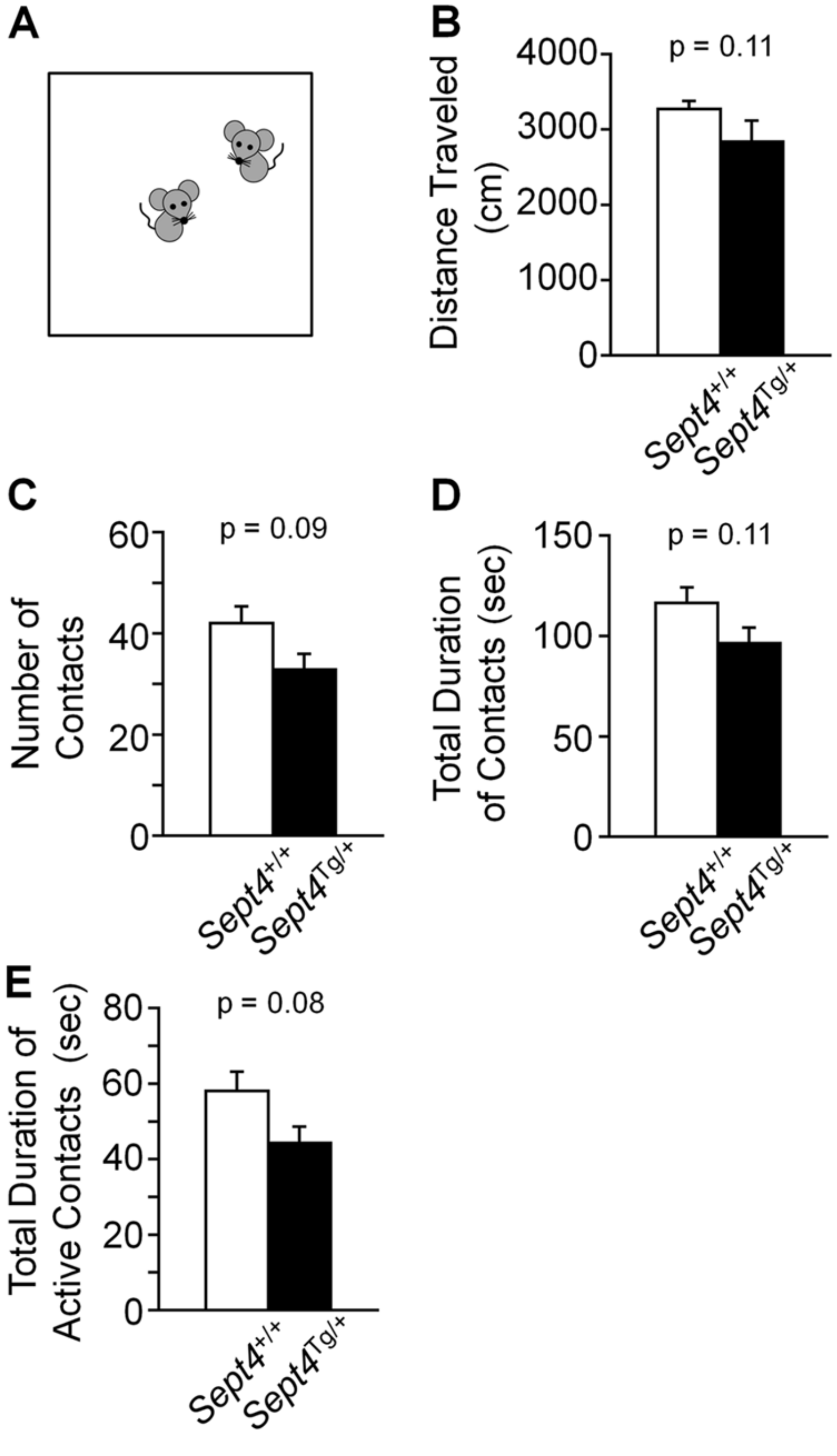

Additional Figure A7. Reduced physical contact between pairs of *Sept4*$^{Tg/+}$ mice in an open field

(A) Social interaction test in a single open field chamber (cf. Figure 6). (B) Distance traveled [$F_{1,14}$=2.90, p=0.11], (C) total number of contacts [$F_{1,14}$=3.43, p=0.09], (D) total duration of contacts [$F_{1,14}$=3.00, p=0.11], (E) total duration of active contacts [$F_{1,14}$=3.44, p=0.08], of *Sept4*$^{+/+}$ and *Sept4*$^{Tg/+}$ mice (n=10 pairs, 7 pairs).

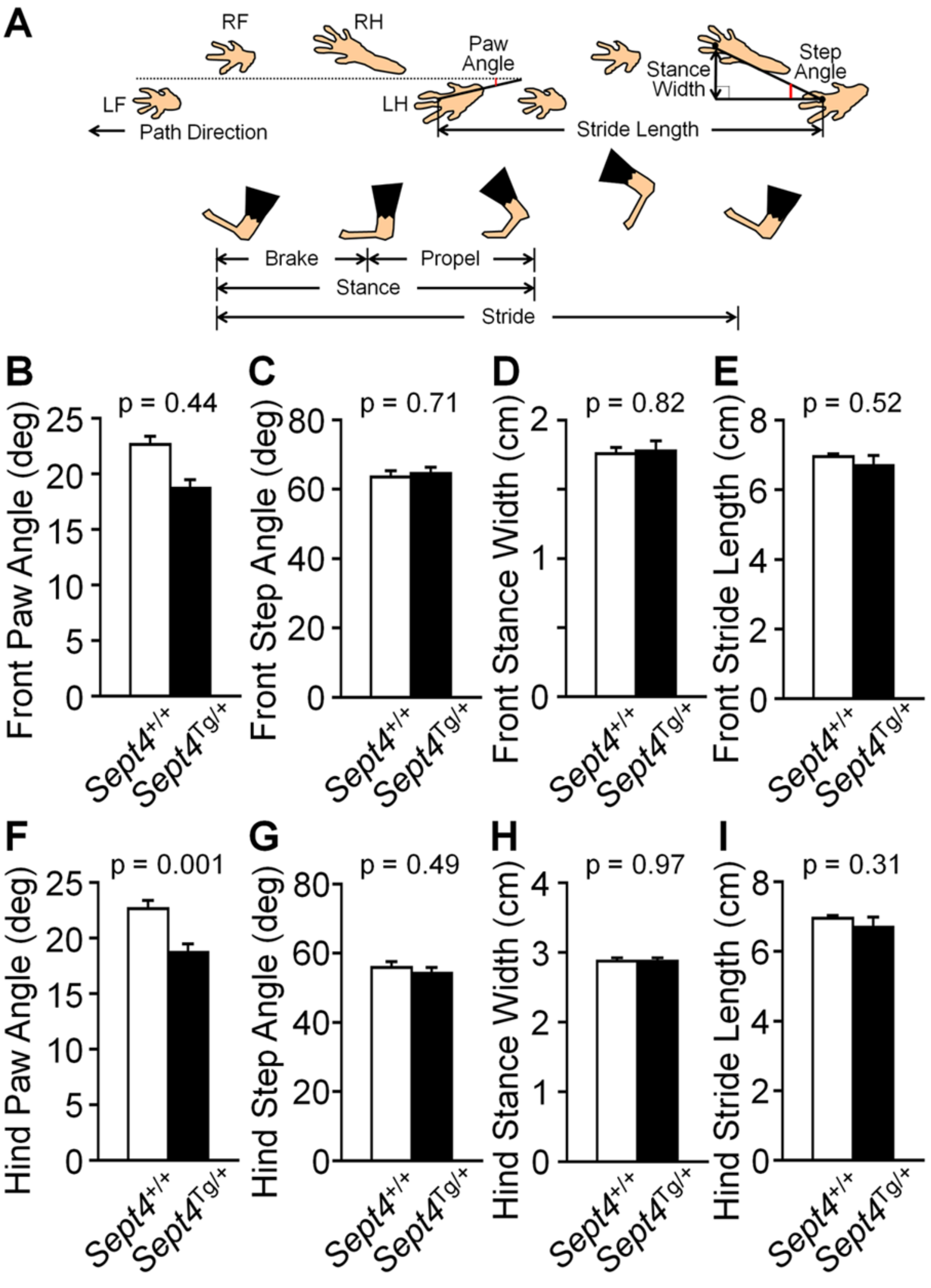

Additional Figure A8. Reduced hind paw splay angle of *Sept4*$^{Tg/+}$ mice in the gait analysis

Narrower paw angles of *Sept4*$^{Tg/+}$ mice (B and F) were the only notable differences among the gait mechanics indices of *Sept4*$^{+/+}$ and *Sept4*$^{Tg/+}$ mice (n=19, 13). Note: Animals with ataxia, spinal cord injury, or demyelinating disease exhibit wider hind paw angles (Powell et al., 1999). [(B) $F_{1,30}$=0.61, p=0.44, (C) $F_{1,30}$=0.15, p=0.71, (D) $F_{1,30}$=0.06, p=0.82, (E) $F_{1,30}$=0.43, p=0.52, (F) $F_{1,30}$=13.09, p=0.001, (G) $F_{1,30}$=0.49, p=0.49, (H) $F_{1,30}$=0.002, p=0.97, (I) $F_{1,30}$=1.06, p=0.31]

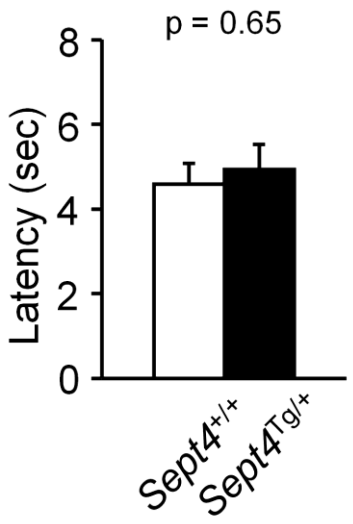

Additional Figure A9. Normal responsiveness of *Sept4*$^{Tg/+}$ mice toward noxious stimuli in the hot plate test

Comparable avoidance responses of *Sept4*$^{+/+}$ and *Sept4*$^{Tg/+}$ mice (n=20, 14) to heat (55°C) given to the paws. [$F_{1,32}$=0.21, p=0.65]

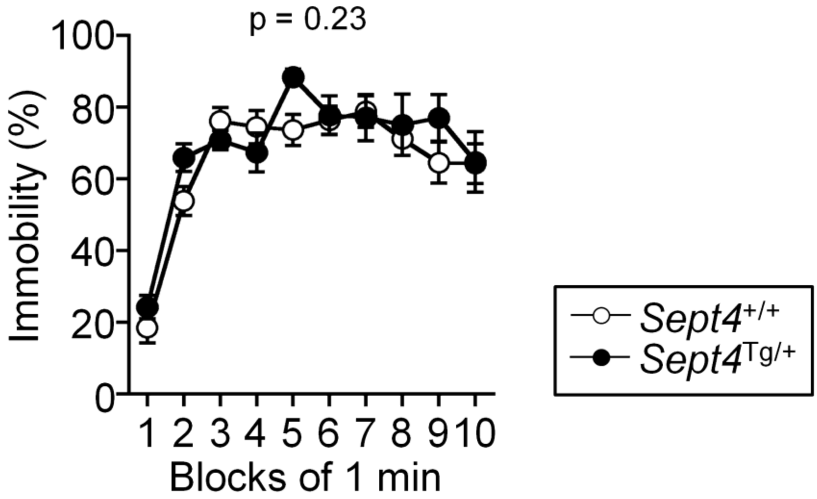

Additional Figure A10. Normal depression-like behavior of *Sept4*$^{Tg/+}$ mice in the tail suspension test

Comparable percent immobility of *Sept4*$^{+/+}$ and *Sept4*$^{Tg/+}$ mice (n=19, 14) suspended in the tail. [$F_{1,30}$=1.50, p=0.23, genotype x block interaction, $F_{9,270}$=1.19, p=0.30]

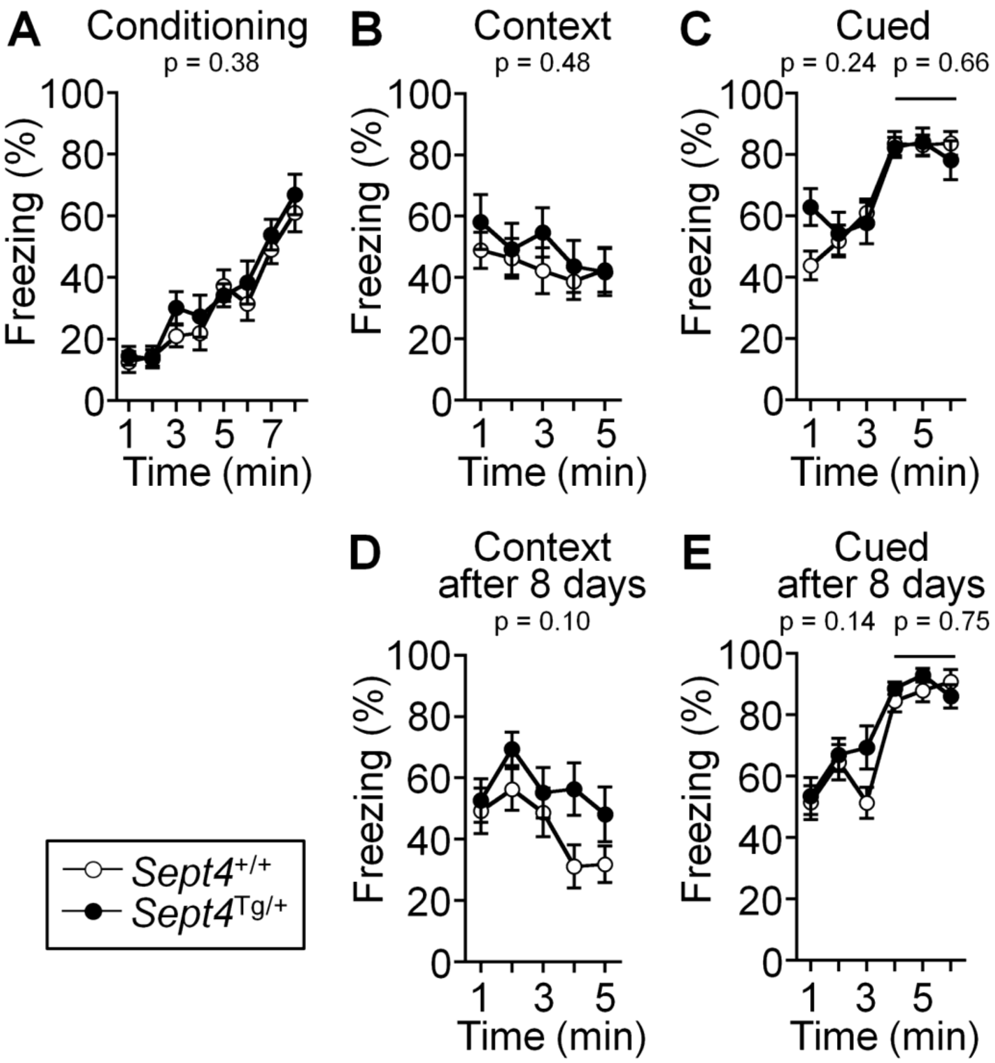

Additional Figure A11. Normal contextual and cued fear conditioning of *Sept4*$^{Tg/+}$ mice
Sept4$^{+/+}$ and *Sept4*$^{Tg/+}$ mice (n=19, 14) exhibited comparable freezing responses (in percent duration) in all of the following indices: (A) Freezing during the acquisition of the association between a 2-second electric shock in the paws and a preceding tone (cue) in chamber A (context). (B and D) Freezing after being housed in chamber A without the tone 1 or 8 days after the conditioning. (C and E) Freezing after the tone in chamber B 1 or 8 days after the conditioning. [(A) $F_{1,31}$=0.79, p=0.38, genotype x time interaction, $F_{7,217}$=0.45, p=0.87, (B) $F_{1,31}$=0.52, p=0.48, genotype x time interaction, $F_{4,124}$=0.46, p=0.76, (C) time 1-3; $F_{1,31}$=1.43, p=0.24, genotype x time interaction, $F_{2,62}$=2.43, p=0.10, time 4-6; $F_{1,31}$=0.20, p=0.66, genotype x time interaction, $F_{2,62}$=0.42, p=0.66, (D) $F_{1,31}$=2.79, p=0.10, genotype x time interaction, $F_{4,124}$=1.13, p=0.34, (C) time 1-3; $F_{1,31}$=2.35, p=0.14, genotype x time interaction, $F_{2,62}$=1.26, p=0.29, time 4-6; $F_{1,31}$=0.11, p=0.75, genotype x time interaction, $F_{2,62}$=3.99, p=0.02]
